# Formation of adherens junctions leads to the emergence of a tissue-level tension in epithelial monolayers

**DOI:** 10.1242/jcs.142349

**Published:** 2014-06-01

**Authors:** Andrew R. Harris, Alicia Daeden, Guillaume T. Charras

**Affiliations:** 1 London Centre for Nanotechnology, University College London, London WC1H 0AH, UK; 2 Department of Physics, University College London, London WC1E 6BT, UK; 3 Engineering Doctorate Program, Department of Chemistry, University College London, London WC1H 0AJ, UK; 4 Ecole Supérieure de Biotechnologie de Strasbourg, Strasbourg, 67400 Illkirch-Graffenstaden, France; 5 Department of Cell and Developmental Biology, University College London, London WC1E 6BT, UK

**Keywords:** Adherens junctions, Desmosomes, Morphogenesis, Tension, Atomic force microscopy

## Abstract

Adherens junctions and desmosomes integrate the cytoskeletons of adjacent cells into a mechanical syncitium. In doing so, intercellular junctions endow tissues with the strength needed to withstand the mechanical stresses encountered in normal physiology and to coordinate tension during morphogenesis. Though much is known about the biological mechanisms underlying junction formation, little is known about how tissue-scale mechanical properties are established. Here, we use deep atomic force microscopy (AFM) indentation to measure the apparent stiffness of epithelial monolayers reforming from dissociated cells and examine which cellular processes give rise to tissue-scale mechanics. We show that the formation of intercellular junctions coincided with an increase in the apparent stiffness of reforming monolayers that reflected the generation of a tissue-level tension. Tension rapidly increased, reaching a maximum after 150 min, before settling to a lower level over the next 3 h as monolayers established homeostasis. The emergence of tissue tension correlated with the formation of adherens junctions but not desmosomes. As a consequence, inhibition of any of the molecular mechanisms participating in adherens junction initiation, remodelling and maturation significantly impeded the emergence of tissue-level tension in monolayers.

## INTRODUCTION

Cells are integrated into tissues by several types of specialised intercellular junctions. Two of these, adherens junctions and desmosomes, serve to integrate the cytoskeletons of constituent cells into a mechanical syncitium (Getsios et al., 2004; Green and Simpson, 2007; Harris and Tepass, 2010), which is key to physiological tissue function. Indeed, in developing organisms, mechanical forces generated by individual cells are transmitted and coordinated along intercellular junctions into tissue-level deformations that drive morphogenesis (Blanchard et al., 2009; Martin et al., 2010). In adult organisms, intercellular junctions endow tissues with the strength necessary to withstand external forces, such as pulsatile fluid shear stresses in blood vessels. In addition to providing mechanical strength, junctions are dynamic, giving tissues fluidity by allowing neighbour exchange during development (Ranft et al., 2010) and collective migration during wound healing (Poujade et al., 2007). Complex signalling pathways regulate intercellular junctions, allowing single cells to organise into tissues (the mesenchymal-to-epithelial transition, MET) or leading to tissue disaggregation (the epithelial-to-mesenchymal transition, EMT) (Thiery and Sleeman, 2006). In particular, METs are involved in the formation of somites (Nakaya et al., 2004), the kidney (Davies, 1996) and the coelomic cavity (Funayama et al., 1999). This great plasticity of tissues has been proposed to play a central role during tumour metastasis where, after emigration from the primary tumour by EMT and passage through the circulatory system, cancer cells can undergo MET to form metastases (Kalluri and Weinberg, 2009).

The biological sequence of events leading to the formation of mature intercellular junctions has been studied extensively (Getsios et al., 2004; Harris and Tepass, 2010). Adherens junctions are assembled through the formation of E-cadherin–catenin clusters following contact between the lamellipodia of two nearby cells. After initial contact, F-actin polymerisation is stimulated next to these clusters, and the junction expands through extended contact of the lamellipodia (Liu et al., 2010; Yamada et al., 2005). Then, the dendritic lamellipodial actin network is remodelled into a peripheral actin belt through the combined action of nucleation by formins (Carramusa et al., 2007; Kobielak et al., 2004) and network rearrangement by myosin contraction and, potentially, α-catenin bundling (Drees et al., 2005; Harris and Tepass, 2010). Later, the actin belt becomes increasingly contractile, a process regulated by the crosstalk between the small GTPases Rac1 and RhoA (Braga et al., 1999; Braga et al., 1997). Desmosomes are formed in temporally distinct stages (Nekrasova and Green, 2013). After initial contact between cells, vesicles enriched in the desmosomal cadherin desmocollin are transported to the cell membrane, followed a short time later by desmoglein-rich vesicles. Concomitantly, desmosomal plaque components (plakophilin, plakoglobin and desmoplakin) are recruited to the membrane to form desmosome precursors. These precursors then become stabilised at the plasma membrane upon interfacing with intermediate filaments to form mature desmosomal plaques (Acehan et al., 2008; Nekrasova and Green, 2013).

In spite of these advances, little is known about how tissue-scale mechanical properties such as stiffness and tension emerge with the formation of intercellular junctions, or to what extent each type of cytoskeletal junction contributes to monolayer mechanics. Here, we monitor the apparent stiffness of epithelial cell monolayers cultured on soft collagen gels using deep atomic force microscopy (AFM) micro-indentation. Time-resolved measurements of the apparent stiffness during monolayer formation, combined with localisation studies and chemical or genetic perturbations, showed that a tissue-scale tension emerged as intercellular junctions reformed and that the emergence of tension correlated with the assembly of adherens junctions but not desmosomes.

## RESULTS

### Deep AFM micro-indentation probes monolayer tissue-level tension

We investigated the mechanical properties of epithelial monolayers growing on soft thick collagen gels using AFM-based micro-indentation. In AFM elasticity measurements, the deflection of a microfabricated cantilever is monitored as its tip slowly indents the sample. This force-indentation data can be fitted to a contact mechanics model to estimate sample elasticity (Fischer-Cripps, 2000). Measurements are typically effected using small indentation depths (<10% of sample thickness) to minimise contributions from the underlying substrate, yielding elasticities of ∼400 Pa for Madin-Darby canine kidney (MDCK) cells growing on glass coverslips (supplementary material Fig. S1A) (Harris and Charras, 2011).

**Fig. 1.**
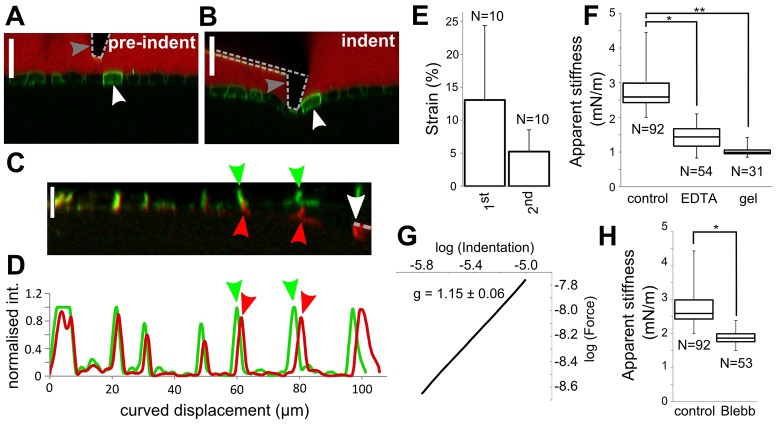
**AFM apparent stiffness measurements are sensitive to the presence of intercellular junctions and reflect the presence of a tissue-level tension in monolayers.** (A,B) Confocal *zx* profiles of a cell monolayer (green) grown on a soft collagen gel (black), before (A) and during (B) indentation with an AFM cantilever (dotted line). White arrowhead, an individual cell; grey arrowhead, the tip of the cantilever. A fluorescent dye was added to the extracellular medium (red). Scale bar: 20 µm. (C) Profile of a monolayer of cells expressing E-cadherin–GFP before (green) and during (red) indentation. Arrowheads, the position of intercellular junctions before (green) and during (red) indentation. White arrowhead, the location of indentation. Scale bar: 10 µm. (D) Fluorescence intensity along a line bisecting the thickness of the monolayer shown in C. Peaks in fluorescence show the position of intercellular junctions before (green line, green arrowheads) and during (red line, red arrowheads) indentation. The cellular strain can be calculated from the change in distance between consecutive junctions along the curvilinear deformation profile (supplementary material Fig. S2A,B). (E) Strain in cells immediately adjacent to the location of indentation (1st neighbours) and one cell diameter further away (2nd neighbours). Data show the mean±s.d. (F) Average monolayer apparent stiffness for control monolayers, monolayers treated with EDTA, and collagen gels without cells. Numbers of individual measurements are indicated underneath each box. (G) Average force–indentation curve collected on mature monolayers plotted on a log-log scale. Axis units are given in log(m) for the *x*-axis and log(N) for the *y*-axis. The curve is an average of 22 individual force–indentation curves. The slope *g* represents the scaling of force with indentation depth. (H) Average monolayer apparent stiffness for control monolayers and monolayers treated with blebbistatin to inhibit myosin activity. Boxes, median, 1st quartile and 3rd quartile; whiskers, maximum and minimum. Numbers of individual measurements are indicated underneath each box. *P and **P<0.01; Student's *t*-test.

We reasoned that by applying deep indentations onto cell monolayers growing on soft collagen gels, we should be able to mechanically deform the cell directly in contact with the cantilever tip, as well as its surrounding neighbours (Fig. 1A–E; supplementary material Fig. S1B). The slope of the acquired force–indentation curves should yield an apparent stiffness for the monolayer-gel composite (supplementary material Fig. S1C) and, if the indentation is sufficiently deep, this mechanical parameter should be sensitive to stretching of the monolayer around the location of indentation. To test this hypothesis, we used MDCK-II cells as a junction-forming epithelial cell model, culturing them on top of thick collagen gels (∼200 µm thick) with an elasticity sevenfold lower than that of the cells (E_collagen_ = 66±8 Pa, E_cells_≈400 Pa; ±s.d.) (Harris and Charras, 2011). We indented monolayer–gel composites using cantilevers with a cylindrical tip to obtain a constant contact area (∼80 µm^2^) that was smaller than the typical cellular apical area (∼300 µm^2^). Indentation depths of 10–15 µm, larger than the monolayer thickness, induced a deformation field that propagated over several cell diameters (Fig. 1A,B, white arrowhead; supplementary material Movie 1; Fig. S1B). Quantification of the strain field away from the location of indentation using confocal *zx* profiles (supplementary material Fig. S2A,B) revealed that the first and second neighbours were significantly stretched by indentation (Fig. 1C–E). Importantly, apparent stiffness, as measured by deep AFM indentation, was sensitive to the presence of intercellular adhesions. We compared the apparent stiffness of control monolayers, the collagen gel alone and monolayers in which intercellular adhesion had been disrupted by EDTA-dependent calcium chelation. Control monolayers grown on gels had an apparent stiffness that was approximately threefold greater than that of the collagen substrate alone (Fig. 1F, K_control_ = 2.8±0.5 mN/m, K_gel_ = 1.0±0.1 mN/m, *P*<0.01; ±s.d.). Monolayers disaggregated by EDTA had a stiffness that was closer to that of the gel and significantly lower than that of control monolayers (Fig. 1F, K_EDTA_ = 1.4±0.3 mN/m, *P*<0.01 when compared with control monolayers). Together with the emergence of a planar strain field in the monolayer in response to indentation (Fig. 1C–E), these data suggested that stresses induced by indentation were transmitted across intercellular junctions. We reasoned that if intercellular junctions propagate stresses, then the radial distance at which the monolayer–gel composite vertical displacement reaches zero should be larger in control monolayers than in monolayers in which intercellular adhesion was disrupted. In *zx* confocal images, the vertical displacement profile had a larger radius in control monolayers than in monolayers treated with EDTA (∼150 µm versus ∼90 µm to reach zero vertical displacement, *n* = 10 curves examined, supplementary material Fig. S2C,D). Taken together, these experiments showed that deep AFM indentation could be used to probe the mechanical properties of monolayers and that these measurements were sensitive to the presence of intercellular junctions.

Based on the difference in elasticity and thickness between the cells and their collagenous substrate, we reasoned that our experiments could be analysed as the indentation of a thin stiff film bound to a soft elastic substrate. Theoretical and computational studies have shown that restoration forces in response to indentation in such composites are dominated by a combination of the film tension and the film elasticity (Zhang and Zhang, 2009). For indentation depths comparable to the film thickness, the film tension dominates, and the applied force increases linearly with indentation depth; whereas for larger depths, film elasticity dominates, and the applied force changes quadratically with indentation depth. Analysis of our experimental force–indentation curves revealed that the applied force scaled linearly with indentation depth (exponent: 1.15±0.06, *n* = 22 curves examined, Fig. 1G; supplementary material Fig. S1C), suggesting that our measurements of monolayer apparent stiffness are primarily influenced by the tension generated by the monolayer. From a biological perspective, this film tension could arise from intercellular tension across junctions, traction stresses transmitted through focal adhesions, or both. Traction force microscopy studies have shown that, in cell doublets, traction stresses initially present at the interface between two cells are redistributed to the doublet periphery, and that the generation of intercellular tension results in an increment in total traction stress (Liu et al., 2010; Maruthamuthu et al., 2011; Mertz et al., 2013). Consistent with the role of contractility in generating both possible sources of tension, the apparent stiffness of monolayers treated with the myosin inhibitor blebbistatin was almost twofold lower than under control conditions (Fig. 1H). Together with analyses of the vertical displacement profile induced by indentation with and without intercellular adhesions (supplementary material Fig. S2C,D), these measurements showed that, in our experimental system, measurements of apparent stiffness are sensitive to the presence of a tissue-scale tension in the monolayer, which is transmitted through intercellular junctions.

### Tissue-level tension increases during the formation of intercellular junctions

**Fig. 2.**
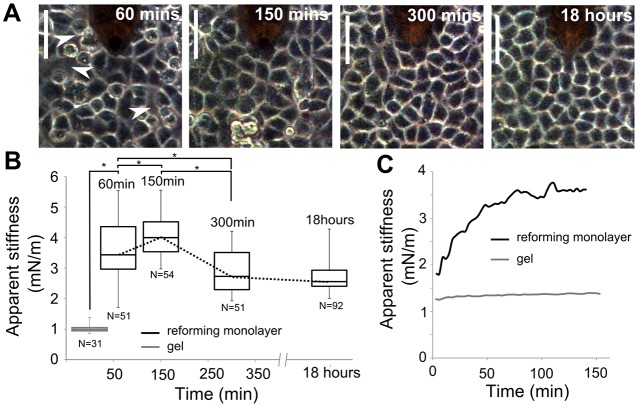
**Apparent stiffness increases in monolayers during the formation of intercellular junctions.** (A) Phase-contrast images of cells reforming a monolayer at different time-points after replating. The AFM cantilever is visible above the monolayer as a dark shadow at the top of the images. Arrowheads, gaps within the monolayer. Scale bar: 50 µm. (B) Temporal evolution of monolayer apparent stiffness obtained from measurements at set time-points. The apparent stiffness of the collagen gel is shown in grey. Dotted line, the temporal evolution of monolayer apparent stiffness. Boxes, median, 1st quartile and 3rd quartile; whiskers, maximum and minimum. The number of measurements for each time-point is indicated below each individual box. *P<0.01; Student's *t*-test. (C) Temporal evolution of monolayer apparent stiffness sampled at 2-min intervals (black line). For comparison, similar measurements were also performed on collagen gels without cells (grey line). The reforming monolayer curve is averaged from three independent experiments.

Using deep AFM indentation, we investigated how monolayer tension evolved during the formation of intercellular junctions. To do this, we seeded dissociated epithelial cells onto collagen gels at densities sufficient to give rise to confluent monolayers immediately upon formation of intercellular junctions. Shortly after seeding, cells started to establish intercellular contacts. By 60 min, cells aggregated into small groups with many gaps subsisting in between (Fig. 2A, arrowheads). After 150 min, a quasi-confluent monolayer had reformed, and the final gaps were being closed (Fig. 2A). By 300 min, the monolayer appeared morphologically indistinguishable from monolayers left to mature overnight (Fig. 2A). Based on the timecourse of monolayer formation observed by phase-contrast imaging, we decided to sample the apparent stiffness of the monolayer at 60 min, 150 min, 300 min and 18 h after replating. In all experiments, the evolution of monolayer apparent stiffness shared the same characteristics; after 60 min, the monolayer apparent stiffness was approximately threefold greater than the stiffness of collagen gels alone (K_60 min_ = 3.6±1.0 mN/m, K_gel_ = 1.0±0.1 mN/m, *P*<0.01), a maximum stiffness was reached at 150 min (K_150 min_ = 4.1±0.8 mN/m, *P*<0.01 when compared with K_60 min_), then the stiffness decreased significantly after 300 min (Fig. 2B, K_300 min_ = 3.0±0.8 mN/m, *P*<0.01 when compared with K_60 min_ or K_150 min_). Monolayers left to grow overnight did not display any further decrease in apparent stiffness (K_300 min_ = 3.0±0.8 mN/m, K_mature_ = 2.8±0.6 mN/m, *P* = 0.06), suggesting that a homeostatic tissue tension had been established by 300 min. To gain further insight into the rapid change observed during the first 200 min after replating, we measured apparent stiffness at 2 min intervals after plating. Two distinct phases could be distinguished in all experiments (Fig. 2C, *n* = 3 experiments, black line); apparent stiffness first increased steadily for ∼120 min before reaching a maximum at ∼150 min after replating. The decrease in apparent stiffness observed after 150 min was intriguing (Fig. 2B). As no gaps were visible between cells after 150 min (Fig. 2A) and cell density increased significantly between 150 and 300 min (supplementary material Fig. S3A), we hypothesised that the increased density might relieve tension and that this could be due to cell divisions (Ranft et al., 2010), neighbour exchanges through which cells minimise intercellular stress (Tambe et al., 2011) or morphological changes accompanying junction maturation. Under our experimental conditions, few cell divisions or rearrangements were observed during the first 300 min (supplementary material Movie 2). During monolayer formation, cell spreading is initially required to establish intercellular junctions; then, as cell contacts mature, they increase in height and cell morphology switches from spread to cuboidal. To investigate whether such morphological changes occurred in our experiments, we measured the temporal evolution of the average cell projected area. We found that the cell area first increased significantly between 60 and 150 min before decreasing significantly after 300 min (supplementary material Fig. S3B, black line; Movie 2). These data are consistent with the idea that the cellular morphological changes that accompany junction maturation contribute to changes in apparent stiffness at long times. As apparent stiffness probes the tension generated by the monolayer, these data suggested that the biological mechanisms underlying intercellular junction formation and maturation might govern the temporal changes in apparent stiffness observed during monolayer formation.

### Tissue-level tension depends on the formation of adherens junctions but not desmosomes

**Fig. 3.**
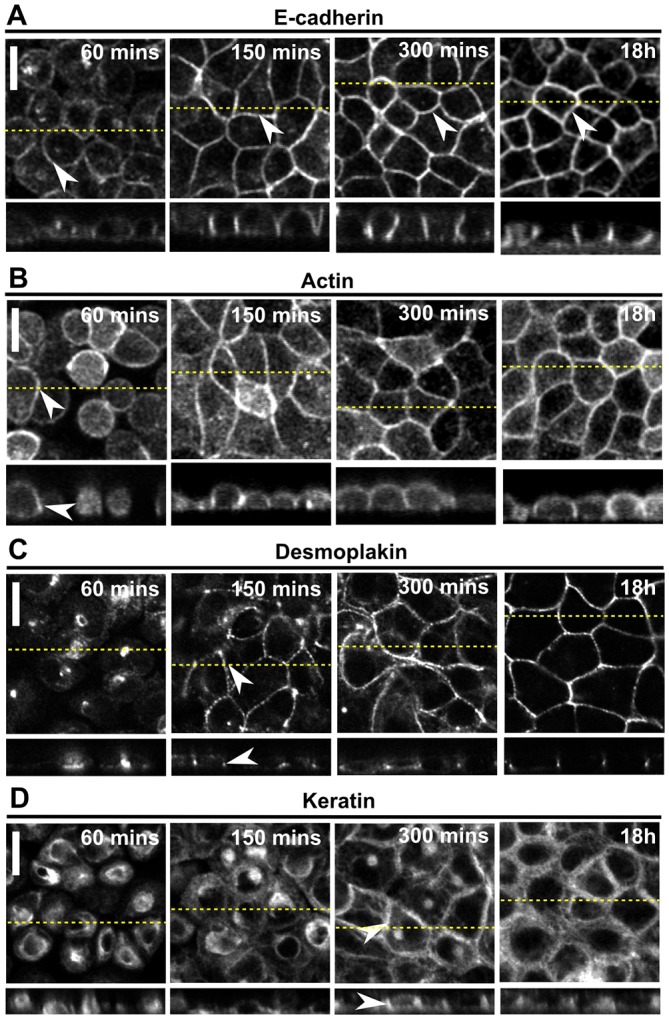
**The timescale for formation of adherens junctions coincides with the establishment of tissue-level tension but the timescale for desmosome assembly does not.** In all panels, the upper images show a single *xy* confocal plane and the lower images show a single *zx* profile. The location of *zx* profiles is shown by dashed yellow lines on the *xy* images, and the position of intercellular junctions is indicated by white arrowheads. The time after replating is indicated on each image. Scale bars: 10 µm. (A) Localisation of E-cadherin–GFP in cells reforming monolayers. (B) Localisation of F-actin in cells reforming monolayers. (C) Localisation of desmoplakin in cells reforming monolayers. (D) Localisation of keratin 18 in cells reforming monolayers.

Next, we sought to correlate the initial observed increase in apparent stiffness with the formation of adherens junctions and desmosomes. The two main components of adherens junctions, E-cadherin and F-actin, localised to cell–cell contacts even at the earliest time-point investigated (Fig. 3A,B, arrowheads). E-cadherin and F-actin localisation was initially limited to the basal side of intercellular contacts (Fig. 3A,B, *zx* profile, 60 min) but from 150 min the height of intercellular junctions increased (Fig. 3A,B, *zx* profile, 150 min) and cell morphology changed from spread to cuboidal (Fig. 3A,B, *zx* profile). The desmosomal plaque component desmoplakin was absent from intercellular contacts at 60 min but gradually localised to junctions over the course of the next 4 h (Fig. 3C, arrowheads), consistent with previous studies (Mattey et al., 1990). Keratin 18 intermediate filaments displayed a perinuclear pattern of localisation, with little or no junctional localisation, for the first 150 min after plating, before gradually acquiring their mature localisation between 150 min and 300 min (Fig. 3D, compare 150 min, 300 min and 18 h). Taken together, these data showed that adherens junctions formed within the first 150 min after plating, coincident with the observed increase in the apparent stiffness of the monolayer. By contrast, the formation of desmosomes and a mature intermediate filament network took significantly longer. Taken together, these mechanical and protein localisation data suggested that the assembly of adherens junctions during monolayer formation led to the observed increase in monolayer apparent stiffness.

**Fig. 4.**
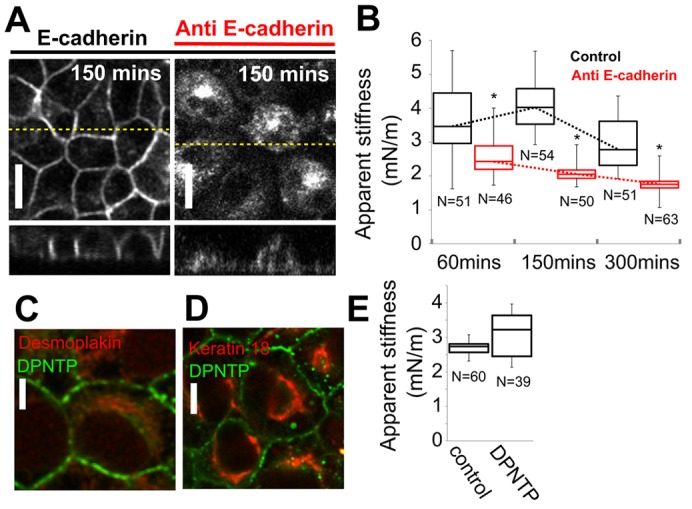
**Contribution of adherens junctions and desmosomes to tissue-level tension.** (A) Localisation of E-cadherin–GFP in control monolayers and monolayers treated with an E-cadherin-blocking antibody. Upper images show a single *xy* confocal plane and the lower images show a single *zx* profile. The location of *zx* profiles is shown by dashed yellow lines on the *xy* images. Scale bars: 10 µm. (B) Temporal evolution of the apparent stiffness in control monolayers (black) and monolayers treated with an E-cadherin-blocking antibody (red). The temporal evolution of monolayer apparent stiffness is indicated by a dotted line for each condition. *P<0.01 between control monolayers and monolayers treated with anti-E-cadherin at a given time-point; Student's *t*-test. (C) Localisation of endogenous desmoplakin (red) in cells expressing the N-terminal portion of desmoplakin tagged with GFP (DPNTP, green). (D) Localisation of endogenous keratin 18 (red) in cells expressing DPNTP–GFP (green). Scale bars: 2 µm. (E) The apparent stiffness of mature control monolayers was not significantly different from that of mature monolayers expressing DPNTP−GFP (*P* = 0.08). In B and E, the number of measurements for each condition is indicated below each box; boxes, median, 1st quartile and 3rd quartile; whiskers, maximum and minimum.

In deep AFM indentation, monolayer apparent stiffness results from a combination of intercellular tension and traction stresses. We focussed our attention on the contribution of intercellular junctions. To investigate the role of adherens junctions and desmosomes, we disrupted the function of E-cadherin and desmoplakin. First, we disrupted E-cadherin-mediated adhesion with a blocking antibody that binds to the ectodomain of E-cadherin (Gumbiner et al., 1988) (Fig. 4A). Under these conditions, cells were able to spread onto the collagen substrate but E-cadherin no longer localised to the cell membrane, and the cells were unable to form intercellular junctions (Fig. 4A). Consistent with our hypothesis, this treatment significantly reduced the increase in apparent stiffness observed following replating (Fig. 4B). Interestingly, in the presence of E-cadherin-blocking antibody, apparent stiffness remained significantly larger than that of the collagen gel, indicating that deep AFM indentation is sensitive to traction stresses. A comparison with control monolayers suggested that traction stresses accounted for at most 50% of the measured increase in apparent stiffness (Fig. 4B). Second, we disrupted desmosomes by generating a cell line stably expressing the N-terminal portion of desmoplakin (DPNTP) tagged with GFP. DPNTP binds to the desmosomal plaque but lacks a keratin-binding domain and, when overexpressed, acts as a dominant negative mutant that prevents the association of the intermediate filament network with desmosomes (Bornslaeger et al., 1996; Huen et al., 2002). Consistent with previous reports, overexpression of DPNTP displaced endogenous desmoplakin from intercellular junctions (compare Fig. 4C with Fig. 3C) and caused keratin intermediate filaments to concentrate in the perinuclear area in mature monolayers [compare Fig. 4D with Fig. 3D (Huen et al., 2002)]. As an intercellular keratin filament network that interfaced with desmosomes formed between 5 h and 18 h after plating (Fig. 3C,D), we reasoned that the impact of desmosome perturbation on monolayer stiffness should be most apparent in mature monolayers plated for 18 h. However, despite the dramatic changes in desmosomal organisation that were induced by DPNTP overexpression, the apparent stiffness of mature DPNTP monolayers was not significantly affected (Fig. 4E, K_Control_ = 2.7 mN/m, *n* = 60; K_DPNTP_ = 2.9 mN/m, *n* = 39; *P* = 0.08). Collectively, these data show that the assembly of adherens junctions coincides with the establishment of a tissue-level tension during monolayer formation and that desmosomes do not play a role in the establishment of tissue tension.

### Actin polymerisation, Arp2/3-mediated actin polymerisation, formin based actin nucleation and myosin contractility are necessary for the establishment of tissue-level tension in monolayers

**Fig. 5.**
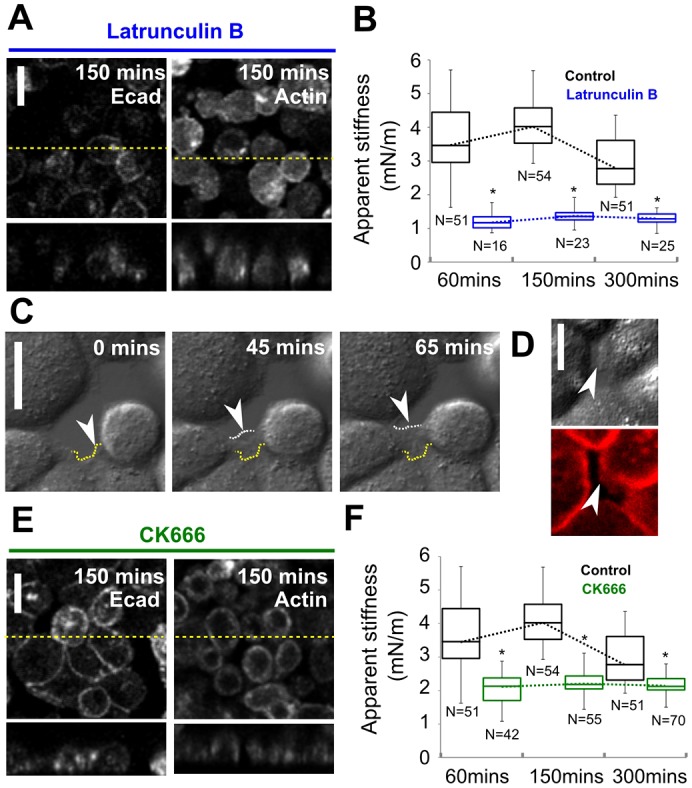
**Inhibition of actin polymerisation and lamellipodial crawling impede the emergence of monolayer tension.** (A) Localisation of LifeAct–Ruby in control monolayers and monolayers treated with latrunculin B 150 min after replating. (B) Temporal evolution of the apparent stiffness of control monolayers (black) and monolayers treated with latrunculin B (blue) to depolymerise the actin cytoskeleton. (C) Differential interference contrast images of cells during monolayer reformation (see supplementary material Movie 3). Cells formed lamellipodia to close existing gaps. Arrowhead, the position of a lamellipodium; yellow dashed line, initial position of the lamellipodium; white dashed line, lamellipodial position at subsequent time-points. (D) Upper image, differential interference contrast imaging of a lamellipodial protrusion during monolayer formation; lower image, phalloidin staining of the same location. Arrowhead, the lamellipodium. (E) Localisation of LifeAct–Ruby in control monolayers and monolayers treated with CK666 to inhibit Arp2/3-mediated lamellipodial protrusion. In A and E, the upper images show a single *xy* confocal plane and the lower images show a single *zx* profile. The location of *zx* profiles is shown by dashed yellow lines on the *xy* images. Scale bars: 10 µm. (F) Temporal evolution of the apparent stiffness in control monolayers (black) and monolayers treated with CK666 (green). In B and F, the number of measurements for each condition is indicated below each box; boxes, median, 1st quartile and 3rd quartile; whiskers, maximum and minimum; dotted line, temporal evolution of monolayer apparent stiffness for each condition; *P<0.01 between control monolayers and treated monolayers at a given time-point (Student's *t*-test).

Next, we examined the role of the biological mechanisms involved in adherens junction formation in the establishment of tissue-level tension. In the current consensus view of adherens junction formation (Harris and Tepass, 2010), lamellipodial crawling, powered by Arp2/3-complex-mediated F-actin polymerisation, allows cells to make initial contacts with their neighbours that can be subsequently broadened by further lamellipodial extension. Consistent with a central role for actin polymerisation, incubating the cells with latrunculin B to depolymerise actin filaments prevented the formation of intercellular junctions and the generation of traction stresses (Fig. 5A). Temporal increases in apparent stiffness following replating were severely diminished (Fig. 5B). Differential interference contrast imaging during monolayer formation (Fig. 5C; supplementary material Movie 3) and phalloidin staining of F-actin (Fig. 5D) confirmed the presence of lamellipodial crawling under our experimental conditions. To examine the role of Arp2/3 in the establishment of tissue tension, we treated reforming monolayers with the Arp2/3-complex inhibitor CK666 (Nolen et al., 2009), which has been shown to inhibit lamellipodial protrusion in neutrophils (Wilson et al., 2013) and aplysia growth cones (Yang et al., 2012). Treatment of mature monolayers with CK666 led to loss of junctional localisation of the Arp2/3 complex (supplementary material Fig. S4D), confirming the effectiveness of the inhibitor in MDCK cells, in agreement with previous work (Tang and Brieher, 2012). When we treated reforming monolayers with CK666, cells no longer formed lamellipodia and this resulted in poorly interconnected monolayers (Fig. 5E). Lack of junction formation and decreased traction stresses severely impeded the establishment of monolayer stiffness (Fig. 5F).

**Fig. 6.**
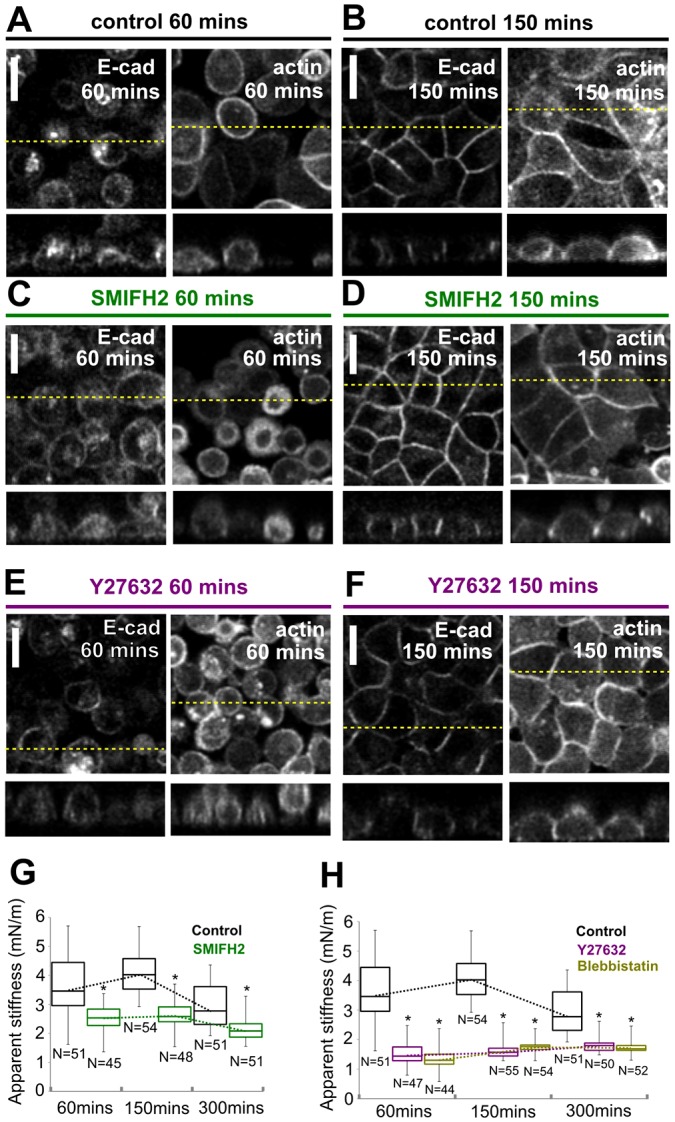
**Actin remodelling by formins and myosin is not required for initial junction formation but is necessary for the emergence of monolayer tissue tension.** (A,B) Localisation of E-cadherin–GFP and LifeAct–Ruby in control cells at 60 min (A) and 150 min (B) after replating. (C,D) Localisation of E-cadherin–GFP and LifeAct–Ruby in cells treated with the broad-spectrum formin inhibitor SMIFH2 at 60 min (C) and 150 min (D) after replating. (E,F) Localisation of E-cadherin–GFP and LifeAct–Ruby in cells treated with the Rho kinase inhibitor Y27632 at 60 min (E) and 150 min (F) after replating. In A–F, the upper images show a single *xy* confocal plane and the lower images show a single *zx* profile. The location of *zx* profiles is shown by dashed yellow lines on the *xy* images. Scale bars: 10 µm. (G) Temporal evolution of the apparent stiffness in control monolayers (black) and monolayers treated with SMIFH2 (green). (H) Temporal evolution of the apparent stiffness in control monolayers (black) and monolayers treated with Y27632 (purple) or blebbistatin (yellow). In G and H, the number of measurements for each condition is indicated below each box; boxes, median, 1st quartile and 3rd quartile; whiskers, maximum and minimum; dotted line, temporal evolution of monolayer apparent stiffness for each condition; *P<0.01 between control monolayers and treated monolayers at a given time-point (Student's *t*-test)

After establishing a sufficiently broad contact, the branched dendritic network of actin is reorganised into a contractile actin belt through myosin contractility and formin-mediated polymerisation of linear F-actin arrays at intercellular junctions (Carramusa et al., 2007; Harris and Tepass, 2010; Kobielak et al., 2004; Tang and Brieher, 2012). Therefore, we investigated the contribution of formins by treatment with the broad spectrum inhibitor SMIFH2 (Rizvi et al., 2009), which has been shown to perturb junctional F-actin in MDCK monolayers (Tang and Brieher, 2012). Upon inhibition of formin activity, intercellular junctions appeared to form normally and some actin remodelling occurred (Fig. 6C,D) but the cells appeared more rounded than those under control conditions after 150 min (Fig. 6B,D). At all time-points, apparent stiffness was significantly lower than under control conditions (Fig. 6G). These results indicated that formin-mediated polymerisation of actin is required for the efficient establishment of monolayer stiffness.

Concomitant with the generation of linear arrays of F-actin by formins, junctional F-actin is also remodelled by myosin activity downstream of Rho kinase (ROCK) (Harris and Tepass, 2010). Hence, we examined the role of myosin contractility in the emergence of tissue-level tension. Upon inhibition of Rho kinase by Y-27632, we observed a reduction in active F-actin remodelling at adherens junctions (Fig. 6E,F), junctions that appeared less taut between cells (Fig. 6E,F) and cells that appeared less spread after 150 min (Fig. 6B,F). Along with these changes, the increase in monolayer apparent stiffness upon junction formation was severely impeded at all time-points (Fig. 6H). Similarly, inhibition of myosin II with blebbistatin abolished the increases in monolayer apparent stiffness that accompanied intercellular junction formation (Fig. 6H), confirming that myosin contractility plays a significant role in setting tissue tension.

### Maintenance of monolayer tension depends on myosin contractility and formins, but not on Arp2/3

**Fig. 7.**
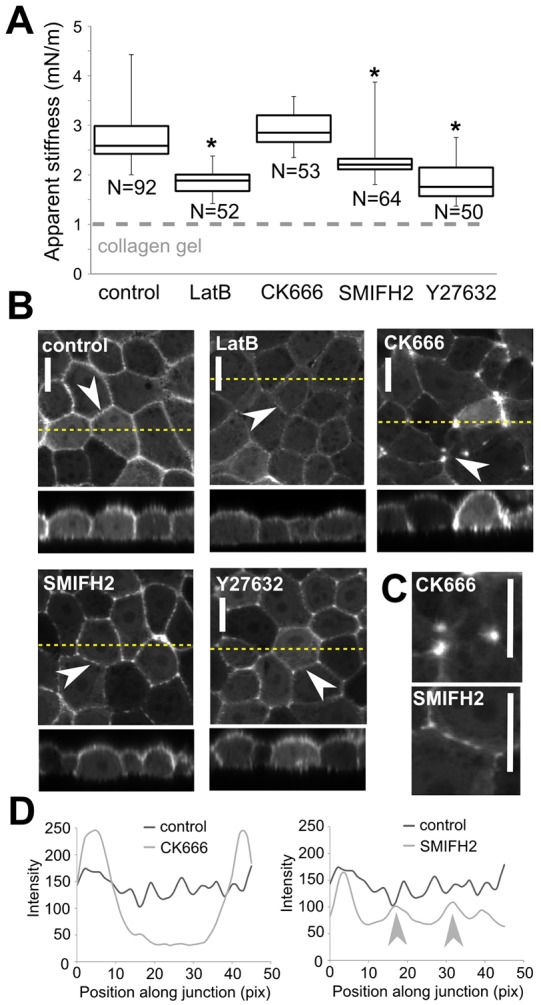
**The effect of chemical inhibitors on tension in mature monolayers.** (A) Effect of chemical treatments on the apparent stiffness of mature monolayers grown under control conditions overnight. The number of measurements for each condition is indicated below each box. Boxes, median, 1st quartile and 3rd quartile; whiskers, maximum and minimum. Dashed grey line, the apparent stiffness of collagen gels. *P<0.01 compared with control (Student's *t*-test). (B) Localisation of LifeAct–GFP in mature monolayers grown overnight under control conditions and treated with chemical inhibitors. Acquisition settings and intensity histograms were identical for all images. The upper images show a single *xy* confocal plane and the lower images show a single *zx* profile. The location of *zx* profiles is shown by dashed yellow lines on the *xy* images. Arrowheads, intercellular junctions. (C) Localisation of LifeAct–GFP along two of the junctions indicated by the arrowheads in B. Scale bars: 10 µm. (D) Fluorescence intensity profiles along the junctions indicated by the white arrowheads for CK666 and SMIFH2 treatments as shown in C. Grey arrowheads, actin enrichment in discrete locations within intercellular junctions of monolayers treated with SMIFH2.

As the biological mechanisms involved in maintaining tensional homeostasis in the monolayer might be different from those leading to the establishment of tension, we examined the impact of cytoskeletal perturbations on the apparent stiffness of monolayers grown overnight under control conditions. Traction force microscopy experiments have shown that, far from the boundaries of cell colonies, traction stresses are small (Mertz et al., 2013); hence, in mature monolayers, intercellular tension should be the main determinant of apparent stiffness. F-actin depolymerisation with latrunculin only led to partial loss of junctional F-actin (Fig. 7B), consistent with previous reports of the existence of a subpopulation of latrunculin-resistant actin filaments at junctions (Abe and Takeichi, 2008; Cavey et al., 2008; Tang and Brieher, 2012; Yamada et al., 2004). Latrunculin treatment was, nevertheless, accompanied by a sharp decrease in monolayer apparent stiffness (Fig. 7A). Treatment of mature monolayers with the formin inhibitor SMIFH2 led to the loss of basal stress fibres, which are assembled from formin-nucleated F-actin (Watanabe et al., 1999) (supplementary material Fig. S4C), consistent with previous results (Tang and Brieher, 2012). SMIFH2 also increased heterogeneity in junctional F-actin fluorescence intensity (Fig. 7B,C). Concomitant with these changes in F-actin organisation, monolayer apparent stiffness decreased significantly (Fig. 7A), consistent with the role of formins in generating linear actin arrays that are necessary to support contractility. Inhibition of Arp2/3 resulted in a decrease in junctional F-actin, an increase in F-actin accumulation at tricellular junctions (Fig. 7B,C) and loss of Arp2/3 localisation to intercellular junctions (supplementary material Fig. S4D), consistent with previous reports (Tang and Brieher, 2012). Despite these changes, Arp2/3 inhibition by treatment with CK666 did not decrease apparent stiffness (Fig. 7A, *P* = 0.029), suggesting that the junctional F-actin network generated by the Arp2/3 complex (Kovacs et al., 2002; Kovacs et al., 2011; Verma et al., 2012) does not participate in the generation of tension in mature MDCK monolayers. Finally, inhibition of Rho kinase by Y-27632 did not significantly affect junctional F-actin appearance (Fig. 7B), but did result in decreased apparent stiffness (Fig. 7A), indicating that myosin contractility is essential to maintain tensional homeostasis in mature monolayers.

## DISCUSSION

**Fig. 8.**
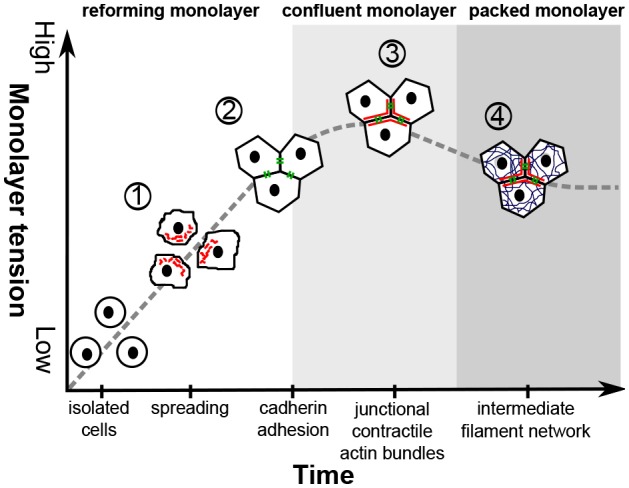
**The emergence of tissue-level tension in monolayers coincides with the formation of adherens junctions.** After replating, cells spread using lamellipodia that result from the formation of a dendritic network of F-actin downstream of Arp2/3 (red, stage 1). Upon the establishment of contact between lamellipodia from neighbouring cells, E-cadherin clusters interface at the membranes of contacting cells (green, stage 2). The dendritic F-actin network is then remodelled at the cell junctions through *de novo* filament polymerisation by formins and myosin-mediated remodelling (stage 3). Later, an intercellular keratin filament network linked by desmosomes is established (blue, stage 4). Monolayer apparent stiffness increases to a maximum after the completion of adherens junction assembly, before decreasing to a lower steady-state tension once monolayers reach homeostasis. Inhibition of each of the molecular mechanisms leading to the formation of adherens junctions perturbs the emergence of tissue-level tension in the monolayer.

Using time-lapse imaging and time-resolved mechanical measurements, together with chemical and genetic perturbations, we have shown that the formation of intercellular junctions is accompanied by an increase in the apparent stiffness of monolayer–collagen composites that reflects the emergence of a tissue-level tension in reforming monolayers; a finding that is relevant to biological processes involving METs. Interestingly, recent traction force microscopy experiments have shown that total traction force increases linearly with the number of cells within colonies (Mertz et al., 2012), suggesting that the steady increase in tissue tension that we observed over the first 150 min after replating reflected a progressive increase in the number of cells interfaced with one another around the location of indentation. The establishment of tissue tension coincided with the assembly of adherens junctions and was sensitive to perturbation of the biological mechanisms involved in their formation (Fig. 8). Furthermore, experiments inhibiting cadherin-mediated adhesion suggested that the formation of intercellular adhesions was the main factor underlying increases in apparent stiffness following replating. Consistent with this, depolymerisation of the actin cytoskeleton inhibited the formation of intercellular junctions, thus abolishing the concomitant increases in monolayer apparent stiffness. Perturbation of the biological mechanisms leading to the assembly of adherens junctions prevented the formation of intercellular junctions and, consequently, the establishment of tissue tension. Furthermore, perturbation of junction maturation after the initial formation of contacts also disrupted the establishment of tissue-scale mechanics. By contrast, during the 150 min timecourse over which the most dramatic increases in apparent stiffness were observed, an intercellular network of intermediate filaments linked by desmosomes did not reform; its assembly after ∼300 min did not correlate with an increase in monolayer tension, and disruption of the interfacing of intermediate filaments with desmosomes did not affect monolayer tension in mature monolayers. As mutations to keratins and desmosomal proteins are known to increase the fragility of tissues (Getsios et al., 2004; Huen et al., 2002), our present results suggest that adherens junctions and desmosomes have distinct mechanical roles, with adherens junctions setting tissue tension and desmosomes governing the maximal deformation a tissue can withstand before failure [i.e. the ultimate strain (Huen et al., 2002)]. Although our study focussed on the role of intercellular junctions, traction stresses applied by individual cells through integrins also contribute to tissue tension, can influence cell–cell tension (Liu et al., 2010; Maruthamuthu et al., 2011) and are likely to be affected by the inhibitors used in this study. Thus, further work will be necessary to fully determine the respective contributions of integrin-mediated traction stresses and tension across intercellular junctions to the evolution of tissue tension during monolayer formation.

Our experimental data showed that apparent stiffness measurements probe the presence of a monolayer tension due to actomyosin contractility. In mature confluent monolayers where traction stresses are low (Mertz et al., 2013), the significant decrease in monolayer tension associated with perturbations to F-actin depolymerisation, myosin contractility and intercellular adhesion, together with the lack of effect of perturbations to desmosomal organisation, argue that tension generated by myosin contractility within individual cells and transmitted through adherens junctions is the main origin of tissue-level tension. These results are in agreement with recent work showing that intrinsic actomyosin activity constitutively exerts tension on E-cadherins at intercellular junctions (Borghi et al., 2012; le Duc et al., 2010; Liu et al., 2010; Maruthamuthu et al., 2011). Intriguingly, in mature monolayers, inhibition of Arp2/3-mediated or formin-mediated actin nucleation had different effects on monolayer apparent stiffness, despite both treatments leading to visible changes in junctional F-actin (Fig. 7D). Indeed, perturbation of formin-mediated actin polymerisation significantly decreased apparent stiffness. By contrast, inhibition of Arp2/3 did not affect apparent stiffness in mature monolayers, in contrast to the results of recent laser ablation experiments that suggest a role for Arp2/3 in junctional tension (Verma et al., 2012). This difference is perhaps due to differences in cell types or incomplete maturation of adherens junctions in gene depletion experiments. Previous work has revealed the presence of two distinct F-actin subpopulations at intercellular junctions, with different turnover dynamics and contractility (Abe and Takeichi, 2008; Cavey et al., 2008; Weber et al., 2007; Yamada et al., 2004; Zhang et al., 2005). These subpopulations are nucleated either by the Arp2/3 complex (Kovacs et al., 2002; Kovacs et al., 2011) or by formins (Carramusa et al., 2007; Kobielak et al., 2004). Furthermore, recent molecular evidence suggests that the function of F-actin networks might be specified during their nucleation, with formins generating linear contractile F-actin arrays (Michelot and Drubin, 2011). Therefore, in light of these studies and our experimental results, we speculate that, in mature monolayers, the F-actin networks generated by Arp2/3 and formins play different mechanical roles, with formins generating a contractile F-actin network that is the major determinant of tissue-level tension. However, further work will be necessary to verify this hypothesis.

Intriguingly, monolayer tension initially increased over the course of 150 min, reaching a peak before decreasing significantly between 150 and 300 min to settle to a steady-state value. This suggested that monolayers might reach tensional homeostasis within ∼5 h. Examination of the temporal evolution of projected cell area and cell density, together with the low frequency of cell division and rearrangements during the first 300 min, leads us to propose that the decrease in tension we observe is mainly due to changes in cellular morphology within the monolayer. Our data suggest that junction-forming cell types adopt different tensional states to reach optimal monolayer configuration when reforming from dissociated cells; first, cells adopt a high-tension state to rapidly assemble intercellular junctions, then, upon reaching confluence, cells optimise their morphology and arrangement within the monolayer to reach tissue tensional homeostasis. This sequence of events is consistent with recent studies showing that mechanical forces applied by myosin contractility within adjacent cells and detected by vinculin and/or α-catenin are necessary for junction maturation in monolayers (Barry et al., 2014; le Duc et al., 2010; Liu et al., 2010; Twiss et al., 2012; Yonemura et al., 2010). Thus, mechanotransductory pathways might play a key role in the evolution of monolayer tension reported in the present study. How the passage from high-tension state to homeostatic-tension state is regulated, what physical parameters are monitored and whether detection is effected at the cellular or tissue level remain unclear and form interesting future research directions with relevance to cancer and development.

## MATERIALS AND METHODS

### Preparation of collagen gels

Collagen gels were made according to manufacturer protocols in a 7∶2∶1 ratio of collagen type 1A (Nitta Gelatin, Japan)∶ 5× DMEM (PAA, Germany)∶ reconstitution buffer (4.77 g HEPES and 2.2 g NaHCO3 in 100 ml of 0.05 M NaOH). The solution was mixed on ice before transfer to 50-mm glass-bottomed Petri dishes (Intracell, Herts, UK) and gelation at 37°C for 30 min. Gels of different thicknesses were made according to the type of experiment. We verified that the elasticity of gels was consistent across all experiments by measuring the elasticity of a control gel devoid of cells prior to each experiment. For confocal imaging, thin gels were generated to accommodate the working distance (∼300 µm) of high-magnification objectives (UPLSAPO, 60× water immersion, NA 1.2, Olympus). To do this, 500 µl of the reconstituted collagen solution was placed into a 50-mm glass-bottomed Petri dish, covering the entire surface of the dish. The majority of the solution was removed to obtain a thin collagen gel (∼100 µm thick), which was left to gel at 37°C. The collagen gel was washed with culture medium and cells were seeded onto it (see replating assay). For AFM measurements, thicker gels were required to reduce any artefactual contribution from the glass substrate during deep indentation. Therefore, 775 µl of collagen gel solution was deposited in the Petri dish and left to gel, giving a gel thickness >200 µm.

### Cell culture

Madin-Darby Canine Kidney II (MDCK-II) cells were cultured at 37°C under an atmosphere of 5% CO_2_ in air in DMEM (Invitrogen, Paisley, UK) supplemented with 10% FCS (Invitrogen) and 1% penicillin-streptomycin. Cells were passaged every 4–5 days using standard cell culture protocols.

### Stable cell lines

To visualise different components of intercellular junctions and the cytoskeleton, we used cell lines stably expressing E-cadherin–GFP, keratin-18–GFP, LifeAct–Ruby (an F-actin marker) and PH-PLCδ–GFP (a membrane marker). The generation of these lines has been described previously (Harris et al., 2012). All cell lines were cultured in the presence of the appropriate selection antibiotics (G418, 1 mg/ml; puromycin, 250 ng/ml). A cell line stably expressing the N-terminal portion of desmoplakin tagged with GFP (Bornslaeger et al., 1996; Huen et al., 2002) was generated by inserting a 1.755-kb fragment from the N-terminal of desmoplakin into EGFP-N1. DPNTP-GFP was then excised from EGFP-N1 and ligated into the pRevTRE retroviral vector (Clontech). Retroviral generation and subcloning were effected as described previously (Harris and Charras, 2011).

### Replating assay

To observe junction reformation, 7×10^6^ cells were plated onto a collagen gel to yield a confluent monolayer without the need for cell division. During the first 30–40 min, cells attached strongly to the collagen gel but did not reform intercellular junctions. Subsequently, intercellular junctions formed. This was imaged by using confocal microscopy, and changes in monolayer apparent stiffness could be measured by using AFM.

### Immunostaining

Collagen gels were coated onto 13-mm glass coverslips. For immunostaining, ∼1×10^6^ cells were plated onto each coverslip and cultured for the required time to reach the desired experimental time-point. For desmoplakin and cytokeratin immunostaining, cells were washed twice with PBS and then fixed in a 50∶50 mix of methanol and acetone at −20°C for 15 min. For ARPC2 immunostaining, cells were fixed with 2% formaldehyde, 0.1% glutaraldehyde and 0.2% Triton X-100 in cytoskeleton buffer (50 mM imidazole, 50 mM KCl, 0.5 mM MgCl_2_, 0.1 mM EDTA, 1 mM EGTA pH 6.8) for 15 min at room temperature. Samples were then incubated with 10% bovine serum albumin (BSA) in PBS for 20 min to block non-specific binding. The mouse anti-desmoplakin-1 and -2 antibody (Progen Biotechnik, Heidelberg, Germany) was used without dilution, the mouse anti-keratin-18 antibody (Abcam, Cambridge, UK) was used at 1∶100 and the polyclonal rabbit anti-ARPC2 antibody (07-227, Millipore) was used at 1∶50. Samples were incubated with each antibody for 1 h at room temperature. After several washes, the sample was incubated with Alexa-Fluor-568-conjugated goat anti-mouse-IgG (1∶100, Invitrogen) or Alexa-Fluor-647-conjugated goat anti-rabbit-IgG (1∶200, Invitrogen) for 1 h at room temperature. Samples were then washed several times with blocking solution before imaging. All immunostaining was repeated at least twice, independently.

### AFM measurements of apparent stiffness

AFM measurements were effected using a JPK CellHesion 200 (JPK, Berlin, Germany). This instrument has an extra-long Z-piezo range (100 µm) enabling force-indentation data to be collected over a large indentation range and ensuring separation of the tip and sample upon retraction. Apparent stiffness measurements were performed on thick gels so that the rigidity of the glass-bottomed dish did not affect measured mechanical properties. Measurements were performed using silicon nitride cantilevers with 20-µm-tall flat cylinder tips (Nanosensors, Neuchatel, Switzerland). The radius of the tip was ∼5 µm, allowing for contact with a whole single cell. The large height of the tip aided in avoiding extraneous contact between the cantilever underside and the cells to yield accurate measurements of the sample stiffness (Harris and Charras, 2011). The spring constant of each cantilever was calibrated prior to experiments (on average k∼0.11 N/m) with the JPK control software.

To measure apparent stiffness, a calibrated cantilever was advanced towards the sample at a constant speed until a user-defined force was reached. Upon contact with the sample, any additional movement of the piezo-electric ceramic resulted in a combination of bending of the cantilever and indentation of the sample. The collected data showed the force versus the distance travelled. The force–distance curve was translated into a force–indentation curve that showed the force needed to result in an indentation of a given depth. The slope of these curves showed the apparent stiffness (supplementary material Fig. S1C). In our experiments, force–distance curves were acquired with an approach velocity of 5 µm/s up to a target force of 25 nN, resulting in indentation depths of ∼15 µm for cells seeded on top of a collagen gel. This indentation depth is approximately twofold larger than the typical cell height and leads to a measurable deformation field in the monolayer. The slow approach speed used was chosen to minimise the contribution of viscoelastic properties of the system to the measured apparent stiffness. Force–distance curves were analysed using custom-written routines in Matlab (Mathworks, Natick, MA) and with the JPK analysis software.

### Measurement of temporal changes in monolayer apparent stiffness

We monitored temporal changes in monolayer apparent stiffness using two different approaches. First, we positioned the AFM cantilever above a chosen area and acquired force–distance curves at 2-min intervals for 3 h using the JPK control software. A phase-contrast image of the cantilever and the monolayer was captured immediately after each measurement. Second, we acquired measurements in multiple positions in a Petri dish at defined time-points after replating (60 min, 150 min, 300 min and 18 h), and returned the monolayers to the incubator between measurements. At each time-point, AFM force–distance curves were collected at five different positions in more than six Petri dishes, giving a total of >30 measurements for each experimental condition. For each time-point, Petri dishes remained out of the incubator for <10 min.

### Measurement of indentation depth and strain field in the monolayer with combined AFM and confocal microscopy

For combined AFM-confocal measurements, the AFM head was interfaced onto an inverted microscope (IX-81, Olympus) equipped with a 60× water-immersion objective (UPLsapo, NA 1.2, Olympus). To image the cantilever shape, an Alexa-Fluor-647-labelled dextran with a molecular mass of 10,000 Da (Invitrogen) was added to the medium and imaged by exciting the dye with a 647-nm laser and emitted light was detected at 680 nm. The cantilever tip appeared as a dark shadow against the bright medium. GFP in the cells was excited with a 488-nm laser and emitted light was detected at 525 nm. The AFM tip was brought into contact with the cell layer and was then lowered to create a ∼15-µm-deep indentation using the AFM stepper motors. The resulting indentation and deformation of the cell layer was visualised by taking an *xz* profile image (with a pixel size of 0.2 µm by 0.2 µm).

### Analysis of cellular deformation profiles resulting from deep AFM indentation

Confocal images were acquired before and during AFM indentation. Images were analysed using custom software written in Matlab. Briefly, images were imported into Matlab and a line profile was drawn that followed the mid-plane of the cells. This line of interest was smoothed and its points interpolated using a cubic spline. The displacement along the line was determined by measuring the Euclidean distance at each pixel. Intercellular junctions along the lines were identified as local maxima in fluorescence intensity along the line profile. The strain in cells within the monolayer could then be measured as the relative change in length between junctions before and during indentation (supplementary material Fig. S2A,B).

### Confocal imaging and image analysis

Confocal images were acquired with an inverted microscope equipped either with an FV1000 confocal head (both from Olympus) or with a spinning disk confocal head (Yokogawa CSU22, Yokogawa, Japan) and an Andor iXon EMCCD camera (Andor, Belfast, UK). Image stacks were acquired with a 60× objective (UPLsapo 60× water, NA 1.2) at 0.6-µm intervals in *z*. Images of monolayers were acquired at set time-points following replating (60 min, 150 min, 300 min and 18 h) to monitor intercellular junction formation. Between image acquisitions, samples were returned to the incubator. Images were analysed with Fiji software (ImageJ, Vale laboratory, CA). For display, the mid-plane image of the cells was selected from the image stack and subjected to a smoothing filter.

### Treatment with inhibitors

Cells were treated by adding the relevant concentration of inhibitor to the Leibovitz L-15 medium in replating experiments. For measurements on mature monolayers, monolayers were incubated with inhibitors for 1 h prior to measurements. For each treatment, AFM force–distance curves were collected from at least six different Petri dishes on at least two different days. In mature monolayers, divalent cations were chelated by addition of 5 mM EDTA (Sigma). To prevent the formation of E-cadherin-mediated adhesion, cells were incubated with 10 µg/ml anti-E-cadherin blocking antibody that targeted the extracellular domain of E-cadherin (Gumbiner et al., 1988) (uvomorulin, monoclonal anti-E-cadherin antibody, Sigma). F-actin was disrupted by incubation with 750 nM latrunculin B (Sigma). To block F-actin nucleation through the Arp2/3 complex and formins, cells were incubated with 100 µM CK666 (Merck Biosciences) and 40 µM SMIFH2 (Merck Biosciences), respectively. To inhibit myosin activity, cells were treated with 50 µM Y27632 (Merck Biosciences) – an inhibitor of Rho kinase – or 100 µM blebbistatin (Merck Biosciences), which inhibits myosin II ATPase.

### Statistics

Values within the text are given as the mean±s.d., and the statistical significance was determined using a Student's *t*-test, where statistical significance was assumed when *P*<0.01. Data in charts are displayed as box and whisker plots in which the box represents the median, 1st quartile and 3rd quartile, and the whiskers represent the maximum and minimum. For all measurements of stiffness, the number of measurements for each time-point is indicated below each individual box, and measurements were taken on at least three separate days.

## Supplementary Material

Supplementary Material
