# Supplementary material for: Formation of adherens junctions leads to the emergence of a tissue-level tension in epithelial monolayers
Source: J Cell Sci. 2014 Jun 1;127(11):2507–17. doi: 10.1242/jcs.142349 (PMC4043320; doi:10.1242/jcs.142349)
Supplement: Supplementary Material [file supp_127_11_2507__index.html]

Formation of adherens junctions leads to the emergence of a tissue-level tension in epithelial monolayers — Supplementary Material 

# Formation of adherens junctions leads to the emergence of a tissue-level tension in epithelial monolayers

## JCS142349 Supplementary Material

**Files in this Data Supplement:**

- **Supplementary Material**
