# Supplementary material for: Formation of adherens junctions leads to the emergence of a tissue-level tension in epithelial monolayers
Source: J Cell Sci. 2014 Jun 1;127(11):2507–17. doi: 10.1242/jcs.142349 (PMC4043320; doi:10.1242/jcs.142349)
Supplement: Supplementary Material [file supp_127.11.2507_JCS142349.pdf]

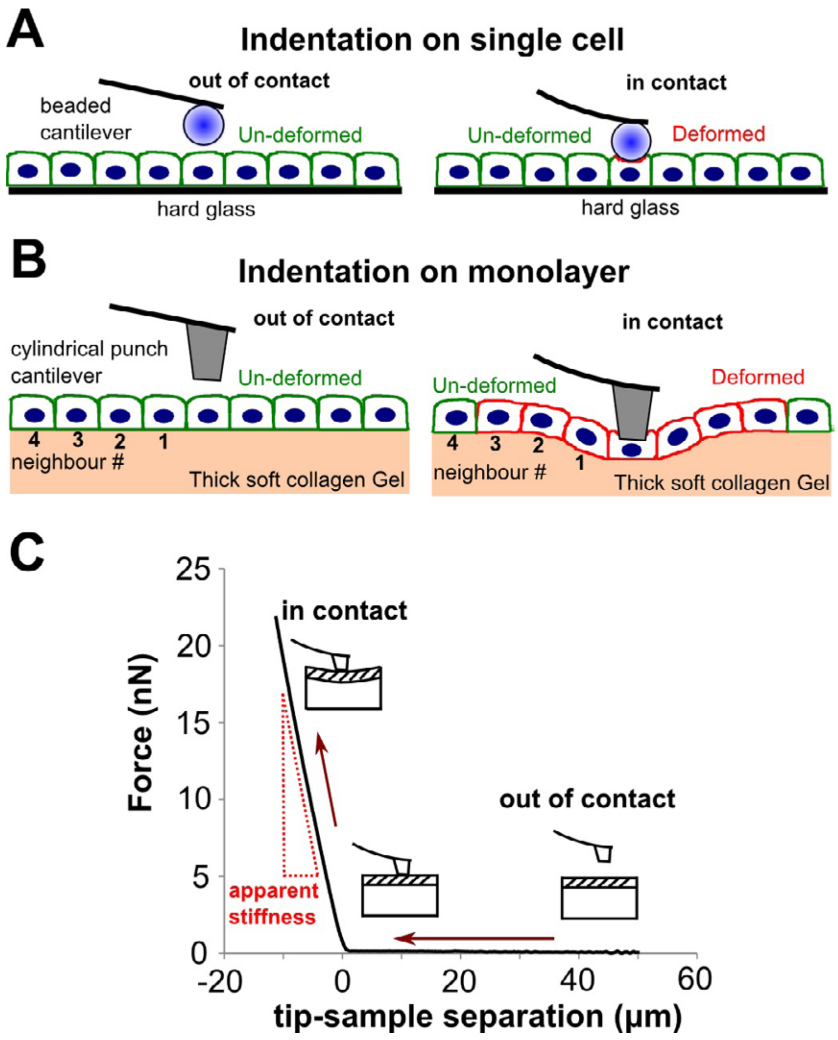

Fig. S1. Experimental setup. (A) In many AFM-based elasticity measurements, samples are indented with a spherical tipped cantilever to a depth of ∼1 µm. This signifies that the induced deformation is limited to one single cell (red, right panel). The cantilever deflection as a function of indentation depth can be fitted with contact mechanics models to measure the elasticity of the indented cell. The small depth of indentation prevents artefacts arising from the rigid glass substrate. (B) To measure the mechanical properties of monolayers grown on gels, indentations were effected using a cylindrical punch tip up to an indentation depth of ∼15 µm (larger than the monolayer thickness). This induces a deformation not only in the cell contacted by the cantilever tip but also in neighbouring cells (red cells, right panel). Therefore, the measured applied force results from a combination of the cell stiffness, the monolayer tissue-scale mechanical properties, and the elasticity of the collagen gel. (C) Experimental force-distance curve collected on a monolayer-gel composite. At the start of approach, the cantilever is far from the sample and it is slowly lowered towards the sample. While it stays out of contact, the force applied stays constant and zero. Upon contact between the AFM cantilever tip and the sample, additional travel of the piezoelectric ceramic results in an increasingly deep indentation and the force applied increases sharply with indentation depth. The monolayer-collagen gel apparent stiffness can be determined from the slope of the force-indentation curve (shown in red). In the pictograms, the white box represents the collagen gel and the hatched box the monolayer.

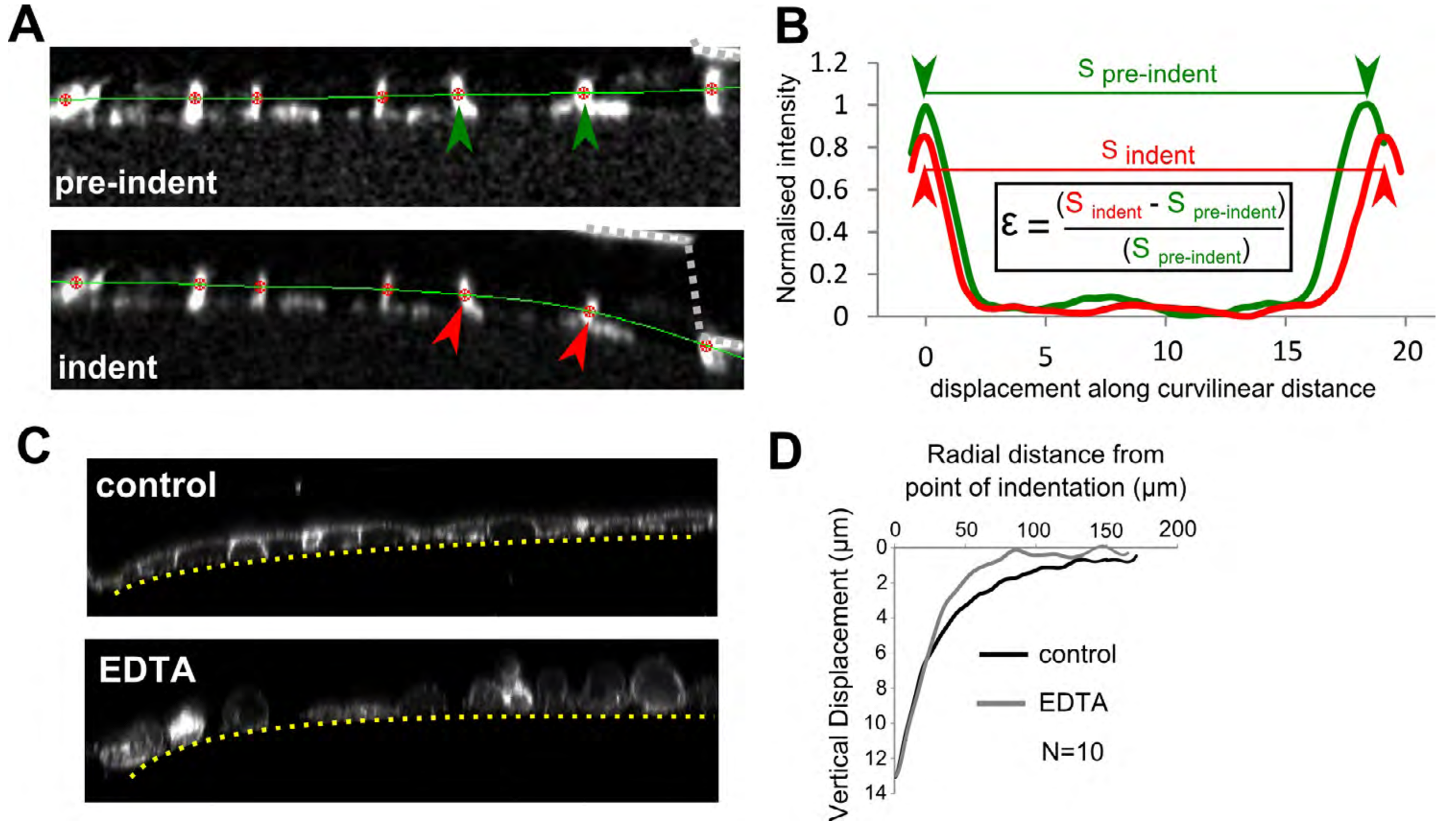

Fig. S2. Analysis of cellular deformation and indentation profiles. (A) The cellular strain field away from the location of indentation was measured by analysing the displacement of intercellular junctions at mid-height in zx profiles of the monolayer using a custom written matlab routine. Confocal zx profiles of monolayers were acquired before (top) and during indentation (bottom). Intercellular junctions could be indentified morphologically as bright vertical bars in cell monolayers expressing E-cadherin-GFP. Indentation took place on the right hand side of the images and the shape of the AFM cantilever tip is indicated by dashed gray lines. The location of intercellular junctions along the line profile (green line) is indicated by red dots. Green arrows indicate two intercellular junctions belonging to one cell close to the centre of indentation before indentation. Red arrows indicate the same junctions during indentation. (B) The GFP fluorescence intensity along the line profile (green line in A) could be compared before (green arrowheads in A, B) and during (red arrowheads in A, B) AFM indentation. Peaks in fluorescence intensity correspond to the location of intercellular junctions and their positions were used to calculate the cellular length (S), the change in cellular length (S_{indent}-$S_{pre\text{-}indent}$) and the engineering strain ($\varepsilon=(S_{indent}-S_{pre\text{-}indent})/S_{pre\text{-}indent}$) along the curvilinear profile. (C–D) In our measurements, increased apparent stiffness concomitant with intercellular junction formation could either be due to an increase in elasticity of the cell monolayer or to the establishment of a tissue-scale tension. One method to determine which of these two hypotheses is correct is to examine the width of the vertical displacement profile generated by indentation to a chosen depth. If the change in apparent stiffness is purely due to an increase in elasticity then the vertical displacement profile should not change when intercellular junctions are disrupted, instead the force necessary to create the indentation should increase (Johnson, 1987). Conversely, if changes in apparent stiffness are due to the generation of tension by the integration of cells into a tissue via formation of intercellular junctions then the width of the vertical displacement profile should decrease significantly for a chosen indentation depth when intercellular junctions are disrupted (Kim and Gouldstone, 2008; Zamir and Taber, 2004). To test this experimentally, we acquired images of the vertical displacement profile for monolayers expressing fluorescently-tagged membrane markers in response to a 15 μm deep indentation before and after disruption of intercellular junctions by calcium chelation with EDTA (C). In both images, the cantilever tip is located at the leftmost extremity of the image. The yellow dotted line delineates the detected vertical displacement profile. (D) Vertical displacement as a function of distance from the location of indentation. In control monolayers, the displacement profile had a width larger than 150 μm (C, *top* and *black curve*, D), whereas EDTA treatment reduced this width to ~90 μm (C, *bottom* and *grey curve*, D). Hence, we concluded that formation of intercellular junctions led to the establishment of a tissue-scale tension rather than an increase in monolayer elasticity, consistent with recent traction force-microscopy experiments examining cell doublets (Maruthamuthu et al., 2011).

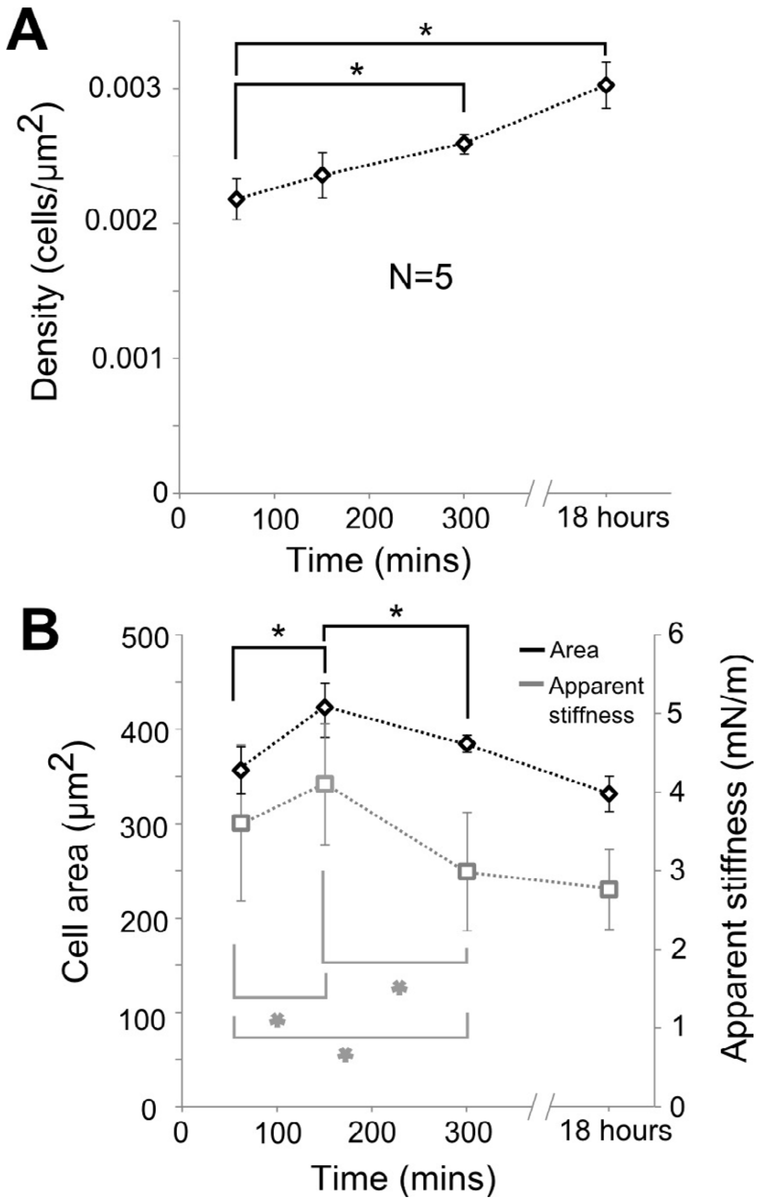

Fig. S3. Evolution of cell area and density over time. In both graphs, asterisks denote significant differences between the indicated time points (*P** <0.01 assessed with a student's *t*-test). (**A**) Temporal evolution of cell density averaged over *N*=5 experiments. Data points are represented as mean±s.d. (B) Temporal evolution of projected cell area (*black line*) and apparent stiffness (*grey line*) over the course of intercellular junction formation and maturation. Data points are represented as mean±s.d. and are averaged over *N*=5 experiments.

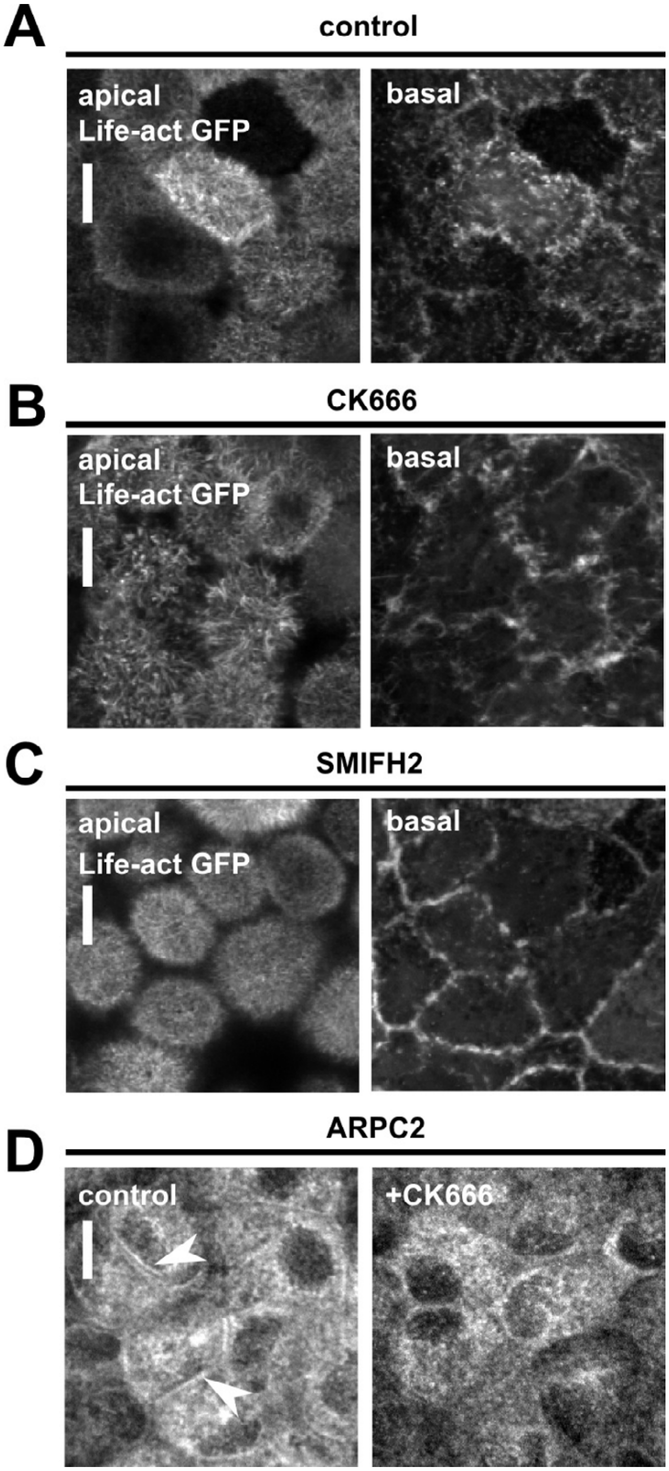

Fig. S4. Effect of perturbations to actin nucleation in mature monolayers grown overnight in control conditions. In panels A–C, images are single confocal sections. In all panels, scale bars=10 µm. (A) F-actin distribution at the apex and base of mature monolayers visualised with Life-Act-GFP. (B) F-actin distribution at the apex and base of mature monolayers treated with CK666 and visualised with Life-Act-GFP. (C) F-actin distribution at the apex and base of mature monolayers treated with SMIFH2 and visualised with Life-Act-GFP. (D) Localisation of the arp2/3 complex at intercellular junctions in mature monolayers treated with DMSO or CK666. Localisation of the arp2/3 complex was revealed by immunostaining against the ARPC2 subunit. Images are a projection of several confocal sections collected from the apical side of intercellular junctions. White arrows indicate intercellular junctions enriched in arp2/3.

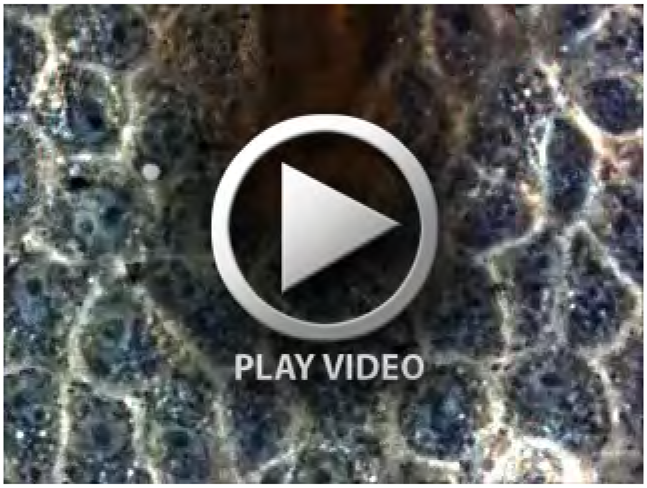

Movie 1. Deformation of the monolayer induced by AFM indentation. Phase contrast movie of AFM indentation of a monolayer grown on a thick collagen gel viewed in the plane. The deformation of the cells surrounding the point of indentation can clearly be observed.

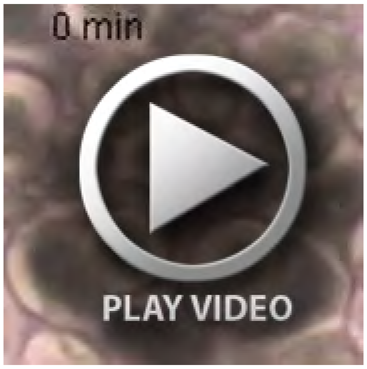

Movie 2. Cell shape change after replating. Phase contrast movie showing the evolution of cell shape after replating cells on a thick collagen gel.

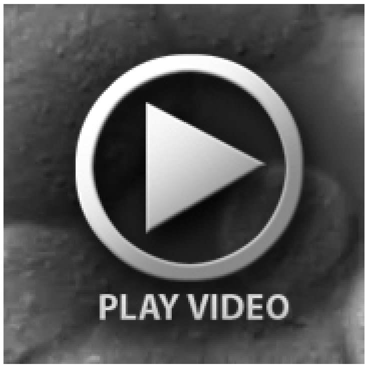

Movie 3. Lamellipodial crawling during gap closure in monolayer formation. Differential interference contrast movie of lamellipodial crawling during gap closure. Lamellipodial crawling enables cells to contact their neighbours and broaden initial intercellular contacts.
